# Science-based policy: targeted nutrition for all ages and the role of bioactives

**DOI:** 10.1007/s00394-021-02662-5

**Published:** 2021-08-24

**Authors:** Alexandre Kalache, Richard P. Bazinet, Susan Carlson, William J. Evans, Chi Hee Kim, Susan Lanham-New, Francesco Visioli, James C. Griffiths

**Affiliations:** 1 International Longevity Centre-Brazil, Rio de Janiero, Brazil; 2 Age Friendly Institute, Boston, MA USA; 3 Department of Nutritional Sciences, University of Toronto, Toronto, ON Canada; 4 Department of Dietetics and Nutrition, University of Kansas Medical Center, Kansas City, KS USA; 5 Department of Nutritional Sciences and Toxicology, University of California, Berkeley, CA USA; 6 Global Government Affairs, Herbalife Nutrition, Los Angeles, CA USA; 7 Nutritional Sciences Department, University of Surrey, Guildford, UK; 8 Department of Molecular Sciences, University of Padova, Padova, Italy; 9 IMDEA-Food, Madrid, Spain; 10 Council for Responsible Nutrition-International, Washington, DC, USA

**Keywords:** Ageing, Diet, Healthy-life-expectancy, Life-expectancy, Nutrients, Nutrition

## Abstract

Globally, there has been a marked increase in longevity, but it is also apparent that significant inequalities remain, especially the inequality related to insufficient ‘health’ to enjoy or at least survive those later years. The major causes include lack of access to proper nutrition and healthcare services, and often the basic information to make the personal decisions related to diet and healthcare options and opportunities. Proper nutrition can be the best predictor of a long healthy life expectancy and, conversely, when inadequate and/or improper a prognosticator of a sharply curtailed expectancy. There is a dichotomy in both developed and developing countries as their populations are experiencing the phenomenon of being ‘over fed and under nourished’, i.e., caloric/energy excess and lack of essential nutrients, leading to health deficiencies, skyrocketing global obesity rates, excess chronic diseases, and premature mortality. There is need for new and/or innovative approaches to promoting health as individuals’ age, and for public health programs to be a proactive blessing and not an archaic status quo ‘eat your vegetables’ mandate. A framework for progress has been proposed and published by the World Health Organization in their Global Strategy and Action Plan on Ageing and Health (WHO (2017) Advancing the right to health: the vital role of law. https://apps.who.int/iris/bitstream/handle/10665/252815/9789241511384-eng.pdf?sequence=1&isAllowed=y. Accessed 07 Jun 2021; WHO (2020a) What is Health Promotion. www.who.int/healthpromotion/fact-sheet/en/. Accessed 07 Jun 2021; WHO (2020b) NCD mortality and morbidity. www.who.int/gho/ncd/mortality_morbidity/en/. Accessed 07 Jun 2021). Couple this WHO mandate with current academic research into the processes of ageing, and the ingredients or regimens that have shown benefit and/or promise of such benefits. Now is the time for public health policy to ‘not let the perfect be the enemy of the good,’ but to progressively make health-promoting nutrition recommendations.

## Introduction

As political, social, cultural, technological, and moral factors change and evolve, concepts and terms can be redefined to reflect the current understanding. “Health” is a term that policy-makers have had difficulty agreeing on its definition in the last century. In 1948, the World Health Organization formulated what seemed to be a ground-breaking definition of health as “a state of complete physical, mental and social well-being and not merely the absence of disease or infirmity.” [126]. In 2011, Huber et al. challenged this definition and proposed a new concept of health as “the ability to adapt and to self-manage, in the face of social, physical and emotional challenges.” [55]. This newer definition is not without criticism and has also been challenged [58].

Regardless of how “health” is defined by policy-makers, today’s consumers want to be empowered to manage their own health, and the increased focus on self-care is often exacerbated by current public health challenges, including non-communicable diseases and/or pandemic. Adoption of a healthy lifestyle, including dietary patterns and when appropriate, oral supplementation, is often included as part of the self-care and self-management tools and interventions to optimize health and nutritional status in individuals at different life stages and facing health challenges and goals [3, 95].

As good nutrition is recognized as a key component of healthy development, well-being, and disease prevention, nutrition research from all sectors is in the quest of understanding what we need to eat to be healthy across the life course. With the advancement of scientific research and as new discoveries are made on the roles of essential nutrients and non-essential nutrients, including bioactives, a newer framework may need to be created and applied so that scientific research can be properly translated to help inform policy-makers to address public health concerns.

This paper is proceedings from the Council for Responsible Nutrition-International (CRN-I)’s 11th Scientific Symposium. The purpose of orchestrating and moderating these annual symposia is to bring current scientific thoughts espoused by academic, industry and policy experts primarily to an audience made up of delegates to the annual Codex Alimentarius (Codex) Committee on Nutrition and Foods for Special Dietary Uses (CCNFSDU). In 2020, the CRN-I symposia occurred as a series of virtual webinars. Previous symposia have focused on optimal nutrition [102], healthy ageing [60, 76], and health promotion [43]. For this paper, academic researchers have presented on the need for adequate protein and amino acid consumption, and the debilitating problem of sarcopenia should adequate intake be compromised. Further, co-authors also consider the intake of other dietary constituents, i.e., omega-3 fatty acids and the pro-hormone, vitamin D and their demonstrated benefits in cognitive development and immunity support, respectively. Oxidative stress and inflammation are blamed for many of the age-related degenerative diseases and the putative role for antioxidant and polyphenolic nutrients to lessen their sequelae was posed. Finally, an end goal would be to propose that regulators consider possible public health objectives to prevent and alleviate the bane of chronic health crises, especially at the end of one’s lifespan.

## The longevity revolution—the WHO decade of health and active ageing

“Revolutions” are events that have a major impact on all sectors of society. “Population Ageing” is a demographic revolution, caused by two main factors: (1) an increase in life expectancy and (2) a decline in fertility rates, and it is now occurring at an unprecedently short period of time. To give an idea of the speed of population aging, the life expectancy at birth in the early 1950s in Brazil was approximately 50 years. Today it is 77 years, a gain of more than 25 years in seven decades of time [73]. Unfortunately, the impact of the COVID-19 pandemic has been particularly disastrous in Brazil and LEB (life expectancy at birth) by the end of June, 2021 has already declined by 3.2 years. In the 1950s, the Brazilian fertility rate was 6.1 where now, like much of the developed world it is below replacement levels [74]. When fertility rates drop below 2.1, the population of parents will not eventually be replaced, a downward spiral on births unable to keep up with deaths [115]. This drop in fertility rates from 6.1 to 1.7 occurred in 50 years [74, 115]. Since 2000, TFR (total fertility rate) in Brazil has been consistently below replacement levels. By 2024 its population will start to decline in common with more than 60 other countries [64].

Until the 1990s, the preponderant view was that ‘Population Ageing’ was strictly a ‘developed world’ issue, with few voices pointing out that it would soon hit the developing world and fast (Table 1). The difference is that while the developed world first became rich and only then gradually aged, in the developing world, ageing is happening at an unprecedented speed while socioeconomic conditions have not improved at the same pace. Even more problematic, with disturbing trends of increasing inequalities.

**Table 1:** Number of persons aged 60 and older in 2017 and estimated at 2050. Note the “percentage change between 2017 and 2050 for Africa, Asia and Latin America compared to the world in toto [114]

	Number of persons aged 60 years or older in 2017 (millions)	Number of persons aged 60 years or older in 2050 (estimated millions)	Percent-age change between 2017 and 2050	Distribution of older persons in 2017 (percentage)	Distribution of older persons in 2050 (estimated percentage)
World	962.3	2080.5	116.2	100.0	100.0
Africa	68.7	225.8	228.5	7.1	10.9
Asia	549.2	1273.2	131.8	57.1	61.2
Europe	183.0	247.2	35.1	19.0	11.9
North America	78.4	122.8	56.7	8.1	5.9
Latin America and Caribbean	76.0	198.2	160.7	7.9	9.5
Oceania	6.9	13.3	92.6	0.7	0.6

In 1982 the United Nations organized the 1st World Assembly on Ageing (WAA) in Vienna, Austria [112]. The resulting report, the ‘Vienna international Plan of Action on Ageing’, was deemed an international framework of standards and strategies and programs with recommendations for action addressing research, data collection and analysis, training and education. However, the report hardly mentions the developing world. By 2002, when the UN organized in Madrid the 2nd WAA [113] the awareness of population ageing as a fully global phenomenon had already taken firm roots, as detailed in the Madrid International Plan of Action on Ageing (MIPAA).

Critical for such change in perception was the pivotal work of the World Health Organization (WHO), through a series of meetings of experts that culminated with the launching of the Active Ageing Policy Framework, which has had a lasting impact on policy development on ageing worldwide [96, 125]. WHO defines Active Ageing as the “process of optimizing the opportunities for health, lifelong learning, participation and security/protection, to enhance the quality of life as individuals and/or societies age”. Thus, it embraces a strong life course approach, which can be encapsulated through the mantra “the earlier you prepare yourself for a long life, the better—but it is never too late”. The Active Ageing Framework not only led to the development of policies in many countries, it also gave birth to enduring international projects, among them the Age Friendly (AF) Cities Guide [127] launched in 2007, which is currently adopted by thousands of cities, with 1350 officially enrolled in the WHO Global Network of AF cities and communities.

The Madrid International Plan of Action on Ageing was focused on three pillars, (1) Older people and development, (2) Advancing health and well-being into old age and (3) Ensuring enabling and supportive environments. Since its launching, the UN Sustainable Goals were adopted and, for the first time ever, ageing was incorporated into the specific Sustainable Development Goals (SDGs) such as Good Health and Well-Being, Gender Equality, No Poverty, Zero Hunger, Reduced Inequalities, etc. Capturing the spirit of the SDGs, the WHO has launched, at the beginning of 2020, with the support of the UN and its specialized agencies, the Healthy Ageing Decade. It encompasses four major areas: (1) Changing how we think, feel and act towards ageing, (2) Strengthening primary health care to be responsive to older people, (3) Create age-friendly cities and communities and, (4) Provide older people who need it access to long term care.

The second major SDG is termed “Zero Hunger”…but it encompasses not only an end to hunger, but just as importantly, achieving food security, promoting sustainable agriculture and improving nutrition. The aged population in both developed and developing countries are very vulnerable to food insecurity. Not only do nutritional needs change as we grow older, but in times of scarcity younger people are often prioritized by families and aid programs. The WHO conclusion is that global nutrition can be achieved by: “(1) making improvements in the nutrient density of food, particularly vitamins and minerals; (2) maintaining intakes of energy and proteins; and (3) recognizing and supporting older people who are poor, isolated and lonely to access healthy meals” [131].

Specific nutrients and bioactives with the ability to improve not only the nutrition of the aged population, but also at any age include, protein and amino acids, omega-3 fatty acids, and Vitamin D.

## The importance of dietary protein and amino acids to the aged population; muscle mass and sarcopenia

A reduction in lean body mass and an increase in fat mass is one of the most striking and consistent changes associated with advancing age. Skeletal muscle [111] and bone mass are the principal (if not exclusive) components of lean body mass to decline with age. These changes in body composition appear to occur throughout life and have important functional and metabolic consequences. The term ‘sarcopenia’ was originally described by Evans and Campbell [30] and further defined [31] as ‘age related loss of muscle mass’. This loss of muscle results in decreased strength, metabolic rate, aerobic capacity and thus, functional capacity. Subsequently, a number of authors have defined sarcopenia more specifically as a subgroup of older persons with low lean body mass (LBM), usually defined as being two standard deviations below the mean LBM of younger persons (usually age 35 years) [4]. Sarcopenia has become recognized as an important geriatric condition and a key precursor to the development of frailty [34, 35, 101]. The original description of sarcopenia was that just as osteopenia (low bone density) predicts risk of a bone fracture, low muscle mass would be a powerful predictor of late-life disability. However, the measurement of muscle mass in humans is difficult with most of the available methods requiring assumptions that may not always be valid and with variable degrees of accuracy and difficulty. In particular, the assessment of total body skeletal muscle mass has, until recently, been problematic. The use of Fat-Free Mass (FFM) as a surrogate assessment of muscle mass has resulted in erroneous conclusions on the importance of skeletal muscle in development of late-life dysfunction and risk of chronic disease.

A meta-analysis [99] of longitudinal observation studies in older people, conducted between 1976 and 2012 (≥ 65 years) examined body composition (bioelectrical impedance analysis (BIA), dual-energy X-ray absorptiometry (DXA) and computed tomography (CT) and physical functional capacity). In the studies that examined lean mass (termed muscle mass) the authors concluded that “low muscle mass was not significantly associated with functional decline.” They also concluded that the role of muscle mass in the development of functional decline was unclear but was “much smaller than the role of fat mass and muscle strength”. These conclusions that the loss of skeletal muscle mass, per se, is only weakly associated with functional outcomes in older people is very likely a result of using the measurement of lean mass as a proxy for muscle mass rather than using a direct measurement of muscle mass.

Indeed, this lack of a strong association between lean mass (as a surrogate measurement of muscle mass) and outcomes in elderly people has led to several consensus definitions of sarcopenia that include measures of muscle strength and/or physical performance in addition to measures of lean mass alone [27]. A project funded by the Foundation for the National Institutes of Health Biomarkers Consortium (FNIH) Sarcopenia Project undertook a data-analysis based effort to better define diagnostic criteria for sarcopenia [107]. Using DXA-derived appendicular lean mass (ALM) from multiple cohort studies in older people, Cawthon et al. [14] described specific cut-points in lean mass that were associated with muscle weakness (grip strength). While the results showed that specific levels of low lean mass were associated with weakness, the association of low lean mass with slow walking speed was inconsistent. Data from the Health, Aging and Body Composition (Health ABC) study showed that strength (grip or quadriceps) but not lean mass (assessed by CT cross sectional area or DXA) was associated with mortality [87]. Although muscle mass was not measured in this study (only lean mass and cross sectional area were assessed), the authors concluded that “Low muscle mass did not explain the strong association of strength with mortality, demonstrating that muscle strength as a marker of muscle quality is more important than quantity in estimating mortality risk”. Because of the weak association between lean mass and outcomes, alternative definitions of diagnostic criteria for sarcopenia have incorporated various measures of strength or function [28, 33, 83, 86]. The consensus definition from FNIH Sarcopenia Project [107] cutpoints for weakness are grip strength < 26 kg for men and < 16 kg for women, and for low lean mass, appendicular lean mass adjusted for body mass index < 0.789 for men and < 0.512 for women.

The D_3_-creatine (D_3_Cr) dilution method uses the assumptions that about 98% of the total body creatine pool is sequestered in skeletal muscle and that creatine is turned over in muscle through the conversion of creatine (Cr) to creatinine (Crn). The additional assumption of this method is that an oral dose of a tracer quantity of deuterated creatine (D_3_Cr) is 100% bioavailable and once absorbed is transported across the sarcolemma and sequestered in sarcomeres. The excretion of Crn and measurement of D_3_-enrichment in urine Crn provides an opportunity to “sample” the intra-myocellular enrichment of D_3_Cr and, thus determine the dilution of the oral label in the whole-body creatine pool in skeletal muscle. Importantly, the measurement does not require dietary control and relies on a single spot, fasted urine sample taken 48–96 h after dosing. The enrichment of urine D_3_Crn reaches isotopic steady-state at about 48 h after dosing and remains stable for about 48 h [22]. Because of this relatively slow turnover of intramyocellular Cr (~ 1.7%/d), subsequent, longitudinal measurements require a pre-dose urine sample as well as a post-dose sample, to correct for residual D_3_Cr. Cross sectional [105] and longitudinal [106] validation studies of this method were performed in rats and in humans.

D_3_Cr muscle mass was strongly associated with whole-body magnetic resonance imaging of muscle mass (*r* = 0.868, *P* < 0.0001) and DXA (*r* = 0.745, *P* < 0.0001). Importantly, the D_3_Cr dilution method measures a parameter, the creatine pool that is associated with skeletal muscle function. In the sarcomere, creatine and creatine phosphate are located adjacent to the Z-disc and the A-band [50] and are not associated with non-contractile components. This strongly suggests that assessment of the creatine pool size provides an indicator of functional muscle mass independent of lipid and fibrotic tissue, both of which increase with advancing age [103] and reduce the accuracy of the measurement of purely anatomic muscle mass using radiographic imaging or using indirect methods like DXA. A particular value of D_3_Cr dilution method is that it can be used in large number of subjects with very little subject burden. This ease of use may allow the D_3_-Creatine dilution method to be used in large subject and patient populations including many where CT or MRI may be difficult such as institutionalized elderly people or infants [32]. For longitudinal trials, there is no limit to how many repeat measurements can be made as long as a urine sample is collected prior to subsequent doses of D_3_-creatine to correct for baseline enrichment of urine D_3_creatinine.

**Fig. 1:**
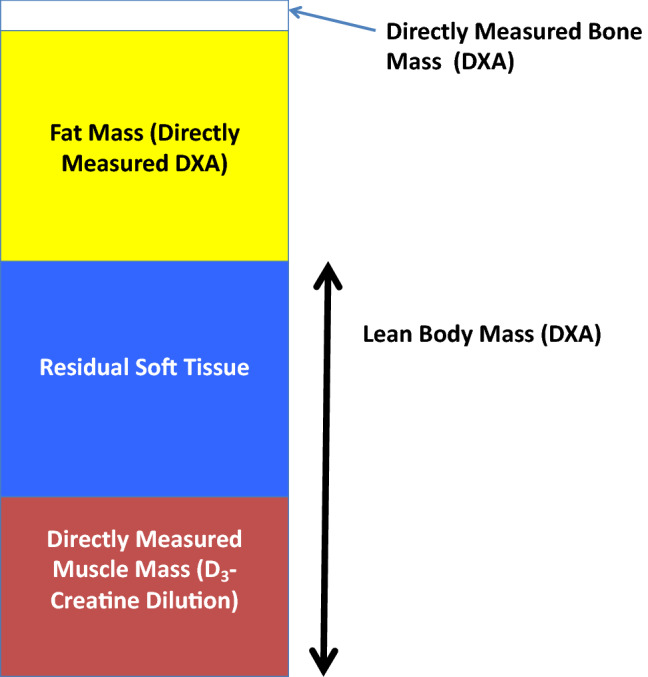
Body composition using DXA and D_3_creatine dilution to measure muscle mass. Importantly, muscle mass is only one component of lean body mass (LBM) which includes blood, viscera, and body water (shown as residual soft tissue). LBM is not a surrogate measurement of muscle mass

Data from the ongoing Osteoporotic Fractures in Men (MrOS) study [15] demonstrated that, in older men, those with the lowest muscle mass/weight by D_3_Cr dilution have the highest risk of incident mobility limitation and injurious falls, and much worse physical performance and lower strength than those with higher muscle mass. These associations were not explained by confounding factors such as age, co-morbidities or activity level. In the same study, DXA based measures of lean mass had much weaker (if any) associations with these outcomes. This is the first large cohort study with a side by side comparison of DXA estimates and D_3_Cr muscle mass measurements on outcomes related to functional capacity and disability. These powerful associations between D_3_Cr dilution estimate of muscle mass and functional capacity and disability provides evidence that D_3_Cr-muscle mass is an assessment of functional muscle mass that is not available using DXA estimates of whole-body lean mass or appendicular lean mass. No significant relationship was observed between muscle mass and appendicular lean mass. In an accompanying editorial, Schaap [100] wrote, “In contrast to DXA, deuterated creatine (D_3_-creatine) assesses muscle mass directly” and “By isolating contractile muscle mass from noncontractile components including fat, the D_3_-creatine assessment is not only an accurate method to assess muscle mass but is less biased by obesity and aging than DX ALM.” In this population, muscle mass was also associated with risk of disability as assessed by activities of daily living and instrumental activities of daily living and mortality [16]. Longitudinal changes in muscle mass in this population were measured by Duchowny et al. [29]. In 40 men (83.4 ± 3.9 years) measured before and after 1.6 years, no change in DXA lean mass, ALM, or body weight were observed. However, the men experienced a significant, average 5.7% (− 1.42 kg) decrease in muscle mass that was accompanied by a reduction on habitual gait speed and grip strength. Without a measurement of muscle mass and using only evidence of no change in LBM, the conclusions on the causes of the longitudinal reduction in function might be quite different. This substantial 3.6% annualized loss of muscle was previously unreported in this age population and points to the need for a better understanding of nutritional strategies as a countermeasure (Fig. 1).

## The bioactivity of omega-3 fatty acids during development

Docosahexaenoic acid (DHA) is an omega-3 fatty acid found in the membrane of all cells, but it is found in particularly high concentrations in the central nervous system, where DHA accumulates rapidly between 22 weeks gestation and 2 years of age [20, 21]. Interest in DHA as a nutrient began with the observation that term-born infants fed infant formulas available in the late 1970s had lower amounts of DHA in their red blood cell membrane phospholipids than did infants fed human milk [90]. Subsequent studies of human milk demonstrated that it contained DHA as well as the omega-6 fatty acid, arachidonic acid (ARA), and established that the red blood cell membrane could be a biomarker (or status indicator) for DHA intake [90]. DHA and ARA were added to most formulas in the U.S. beginning in 2002 after it was reported that infants dying accidentally, who were fed formulas without DHA and arachidonic acid had lower accumulation of DHA in their frontal cortex than infants fed human milk [75]. Other early work in preterm and term infants showed benefits of higher cortical visual acuity, and very early work in preterm infants found evidence of improved cognitive function [124].

It eventually became evident that human milk DHA content was largely dependent upon maternal DHA intake (although women with some alleles in fatty acid desaturases do not appear to efficiently transfer DHA to their milk). Similarly, we learned that DHA and ARA are preferentially transferred across the placenta, meaning that maternal DHA is important to fetal as well as neonatal DHA accumulation [46]. With optimal intake, the majority of DHA accumulates in adipose tissue laid down in the last trimester of fetal life and this has been suggested to be a store of DHA for postnatal development [45]. Numerous other studies showed DHA synthesis from α-linolenic acid could not substitute for DHA intake to improve DHA status. Together these observations form the basis for considering DHA as a conditionally essential nutrient for optimal human development. Our group and others have been studying this question for many years.

One group has focused largely on the question: Is DHA a conditionally essential nutrient for cognitive development? Pre-clinical work with nonhuman primates found that reducing brain DHA during development resulted in poorer cortical visual acuity and cognitive function provided plausibility [85, 93]. In the past 20 years, this question was addressed in RCTs that provided DHA and ARA postnatally and prenatally. The postnatal study began in 2002 and included a group of infants fed formula without DHA and ARA, which we would not consider ethical today. The study was designed for the primary purpose of evaluating cortical visual acuity in infants exposed to none or one of three amounts of DHA in infant formula, however, their cognitive development was followed out to age 6. That prenatal study began in 2006, after most infants in the U.S. were receiving DHA and ARA postnatally either from human milk or supplemented formulas. In both studies, we evaluated cognitive development using the same tests and ages [23, 24].

The cognitive outcomes chosen were age-appropriate and targeted to measure specific cognitive functions as well as studies of verbal and full-scale IQ (Table 2).

**Table 2:** Measurement of specific cognitive functions compared to age in months

Task	Age (months)											
	1	4	6	9	12	18	36	42	48	60	66	72
Visual acuity	•	•		•	•							
Visual habituation		•	•	•								
Bayley scales of infant development (II)						•						
Stroop tasks							•	•	•	•		
Dimensional change card sort							•	•	•	•		
Peabody picture vocabulary test (PPVT)										•		
Electrophysiology (ERP): Go/No Go Tasks											•	
Weschler preschool intelligence scale (WPPSI: IQ)												•

The primary outcome of the postnatal, dose–response study of DHA showed that visual acuity was enhanced by supplementation with DHA and ARA, however, all doses were equivalent in benefit [9]. In age appropriate tests of inhibition, all supplemented groups scored above the control group, however, not all doses achieved significance for all cognitive measures tested. Higher verbal IQ at 5 and 6 years was found using the Peabody Picture Vocabulary Test (PPVT) and Weschler Preschool Primary Scale of Intelligence (WPPSI), respectively, in the combined supplemented groups compared to the control [23]. Go/No-Go tasks at 5.5 years (using an electrode array) [68] and 9 years (MRI) showed faster processing speed with DHA and ARA [67]. Postnatal supplementation enhanced synchrony of neuronal networks. At 9 years, the parietal regions (attention) and anterior cingulate cortex (inhibition) regions of the brain showed greater activation to incongruent compared to congruent trials in the supplemented groups [67].

No significant benefit of prenatal DHA supplementation (placebo vs 600 mg DHA/day) on cognitive outcomes after 3 years of age was recorded. However, the maximum amplitude difference of children correctly completing the Go/No-Go task at 5.5 years was greater in mid-brain (the visual processing that occurs when children are presented with the Go/No-Go stimuli) in both male and female children whose mothers were assigned to DHA. Boys whose mothers received DHA compared to the placebo group also showed a greater difference in amplitude in the frontal cortex while girls showed a significant difference in amplitude regardless of maternal DHA supplementation in this test of inhibition. The amplitude in both the prenatal placebo and DHA groups was much higher than the control group in our postnatal study, possible due to the fact that this group received neither prenatal nor postnatal DHA supplementation [44].

In addition to cognitive benefits of prenatal supplementation and evidence of differences in brain function in both prenatally and postnatally DHA exposure children, other benefits related to prenatal supplementation (despite what might be considered good postnatal supplementation) became apparent, including a reduction in early preterm birth (< 34 weeks gestation) [13, 49], higher fat free mass at age 5, and protection against increased blood pressure related to overweight (BMI > 85th%ile) in children 4–6 years of age (longitudinal assessments every 6 months) whose mothers were supplemented with DHA compared to overweight children whose mothers received the placebo [61].

## Vitamin D and immunity: current controversies and future perspectives

Vitamin D is a truly unique nutrient: it is not a ‘vital-amine’ in the true sense of the word but rather a ‘pro-hormone’ which is produced in the skin during exposure to sunlight (Ultra-Violet B [UVB] radiation at 290–315 nm) [117]. It is now well-established that during the winter months in areas of Northern Latitude, the solar elevation remains low throughout the short daylight period, and there is insufficient solar UVB to support significant vitamin D synthesis. However, it is important to note that UVB induced vitamin D production is not ‘zero’ in the winter since there is evidence of extra-renal vitamin D synthesis [51].

Vitamin D is the generic term for two molecules: namely ergocalciferol (vitamin D_2_) and cholecalciferol (vitamin D_3_). Ergocalciferol is derived by UVB irradiation of ergosterol, which is found in fungi and plants. Cholecalciferol is formed from the effect of UVB irradiation on the skin as discussed above. The action of sunlight on the skin converts 7-dehydrocholesterol to previtamin D, which is metabolized to vitamin D by a temperature-dependent isomerization. Vitamin D is then transported via the general circulation to the liver, where the enzyme 25-hydroxylase converts it to 25 hydroxy-vitamin D (25 OHD). The kidney is the site for further conversion to the active metabolite known as 1,25-dihydroxyvitamin D (1,25 (OH)_2_D_3._) [110].

It is well-established that 25 hydroxy-vitamin D is the best indicator our nutritional, clinical status and is considered the key circulating vitamin D metabolite. Calcitriol (1,25 (OH)_2_D_3_) is the active form of the vitamin, involved in calcium (Ca) homeostasis. Calcitriol helps to maintain normal blood levels of Ca and phosphorus and promotes Ca absorption and bone mineralization, which is why the importance of vitamin D to human and animal health has traditionally always centred around musculo-skeletal integrity [11].

The World Health Organization have reported that there is a relatively high prevalence of poor vitamin D status globally, including populations living in low latitude areas where there is often an abundance of sunlight [52] The reason for this is multi-factorial; including environmental factors (such as air pollution) and cultural aspects which may result in skin not being exposed to sunlight. It is also well-established that the elderly (especially those house-bound) are at a special risk of low vitamin D status. Vitamin D status is reflected by the level of the circulating metabolite 25-hydroxyvitamin D (25OHD), which is produced by hepatic hydroxylation of vitamin D coming from either skin or the gut from dietary intake or vitamin D supplements. A low 25-hydroxyvitamin D level (as defined in the UK by a 25OHD concentration of < 25 nmol/l [118]; and in the U.S. and other countries by a 25OHD concentration of < 30 nmol/l) [88] suggest that vitamin D stores in the body are depleted and concomitantly, vitamin D-requiring functions may be somewhat impaired.

Vitamin D (via the actions of vitamin D active metabolites) regulate over two hundred genes in the body, and this includes those genes which are specifically responsible for cellular proliferation, differentiation and apoptosis [53] What has been intriguing to the vitamin D field, is the discovery of the expression of nuclear vitamin D receptors (VDR) [62] and vitamin D metabolic enzymes in immune cells [63]. This identification provides strong scientific evidence for the potential role of vitamin D in maintaining immune homeostasis and in preventing the development of specific autoimmune processes.

It has been hypothesised that there is an association between seasonal upper respiratory infections and poor vitamin D status because both seem to occur in the autumn/winter months [41] However, controversy remains as to whether there is a direct link between the seasonality of influenza and vitamin D deficiency. It is important to note that the increased influenza incidence often seen in the autumn and winter may be due to behavioral reasons such as a longer time spent in indoor areas, which increases the closeness of individuals and subsequently the likely inter-personal transmission.

There is observational evidence to show that vitamin D inhibits pulmonary inflammatory responses, whilst at the same time enhancing innate defense mechanisms against respiratory pathogens [56]. Furthermore, there are population-based, cross sectional studies which indicate positive associations between circulating 25OHD status and markers of and lung function [39]. However, data are limited, and further research is required in this area, particularly with randomized controlled trials (RCTs).

Upper and acute respiratory tract infections (URTI/ARTI) are the most common of infectious diseases and it is now considered that over two hundred different viruses contribute to specific clinical symptoms [108]. In the first of two comprehensive systematic review and meta-analyses of individual participant data from vitamin D supplementation RCTs, vitamin D supplementation was shown to reduce the risk of ARTI, with the greatest benefit in those with vitamin D deficiency (25OHD status < 25 nmol/l) at baseline [77]. However, it is important to note the limitations to this systematic review/meta-analysis in that there was a high level of heterogeneity in the findings and the clinical definitions of ARTI varied across the studies included in the analysis, with many research participants with ARTI being self-diagnosed. In addition, the results from the meta-analysis of the twenty-four studies included was dependent on the inclusion of two studies in developing countries (Mongolia and Afghanistan). It has been postulated that findings from subjects from developing countries should not be extrapolated to populations from more developed countries, as the efficacy of many treatments may be substantially greater in those developing countries. However, in the most recent systematic review and meta-analysis just published from the same group, and now involving over forty-two trials, there is far less dominance of the findings from developing countries [59]. What is particularly striking is that the vitamin D studies that were most effective in reducing ARTI gave low doses of vitamin D (400–1000 IU/d) on a daily basis; the large bolus doses were not as effective, with many studies showing no significant effect. Further research is urgently required, particularly in ethnic minority groups [48].

There has been, over the last 12 months, much interest in the potential link between vitamin D and the incidence of the SARS-CoV-2 virus, and the disease COVID-19 that is caused by SARS-CoV-2, with calls for widespread high dose vitamin D supplementation for populations. In the observational studies published to date, it is key to note that vitamin D is a negative acute phase reactant, and hence vitamin D status will decline upon infection; hence this does not necessarily mean that it is in the causal pathway. (1) Studies investigating vitamin D and COVID-19 are underway currently but no clear evidence from vitamin D RCTs is, as yet, emerging. Since ethnic minorities are disproportionately affected with Covid-19, further research is certainly justified given the abundance of data showing extensive vitamin D deficiency in these ethnic groups [65].

The U.S. Department of Agriculture publishes the quinquennial Dietary Guidelines for Americans (now in the 8th edition, 2015–2020). Their work details nutrients that are underconsumed such that current intakes may pose a substantial public health concern. Data on nutrient intake, corroborated with biochemical markers of nutritional status where available, and association with health outcomes are all used to establish a nutrient as a “nutrient of concern”. Underconsumed nutrients, or “shortfall nutrients,” are those with a high prevalence of inadequate intake either across the U.S. population or in specific groups. Of the underconsumed nutrients, calcium, potassium, dietary fiber, and **vitamin D** (emphasis added) are considered nutrients of public health concern because low intakes are associated with health concerns [116].

## Diet and bioactives in the management of inflammaging

Inflammation and its sequelae play major role in the onset and development of degenerative diseases, including atherosclerosis, cardiovascular disease, cancer, and neurodegeneration. For the sake of brevity, inflammation can be classified into two main categories: (1) acute and (2) low-grade, chronic inflammation [10]. The inflammatory response is beneficial as an acute, transient reaction to harmful conditions, facilitating the defense, repair, turnover and adaptation of many tissues. However, chronic and low grade inflammation, also termed ‘inflammaging’ due to the manifest changes at the physiologic level, might be detrimental for many tissues and for normal functions [76, 134]. How can we discriminate the two types of inflammation? The acute one usually exhibits the classic semeiotics of rubor, tumor, calor, dolor, and function lesa^1^ [19, 47]. It can be treated with an array of anti-inflammatory drugs such as steroidal (e.g., cortisone) or nonsteroidal (e.g., acetylsalicylic acid or ibuprofen or non-steroidal anti-inflammatory drugs (NSAIDs)). Chronic inflammation is subtler and should be tackled by appropriate lifestyle interventions (see below).

The first one to strongly suggest an etiological link between inflammation and atherosclerosis was the late Dr. Russel Ross, whose 1999 paper in the *New England Journal of Medicine* [97] paved the road for subsequent investigation [40]. It took researchers many years to clinically prove that link, which was finally confirmed—as a proof-of-concept—by the CANTOS study [1]. An association between inflammation and poor cancer prognosis is also well-established (and further confirmed by the CANTOS data [78, 94], whereas we only have plenty, yet suggestive, i.e., not clinically proven, evidence linking low-grade inflammation with neurodegeneration, including cognitive decline or dementia [12].

**Fig. 2:**
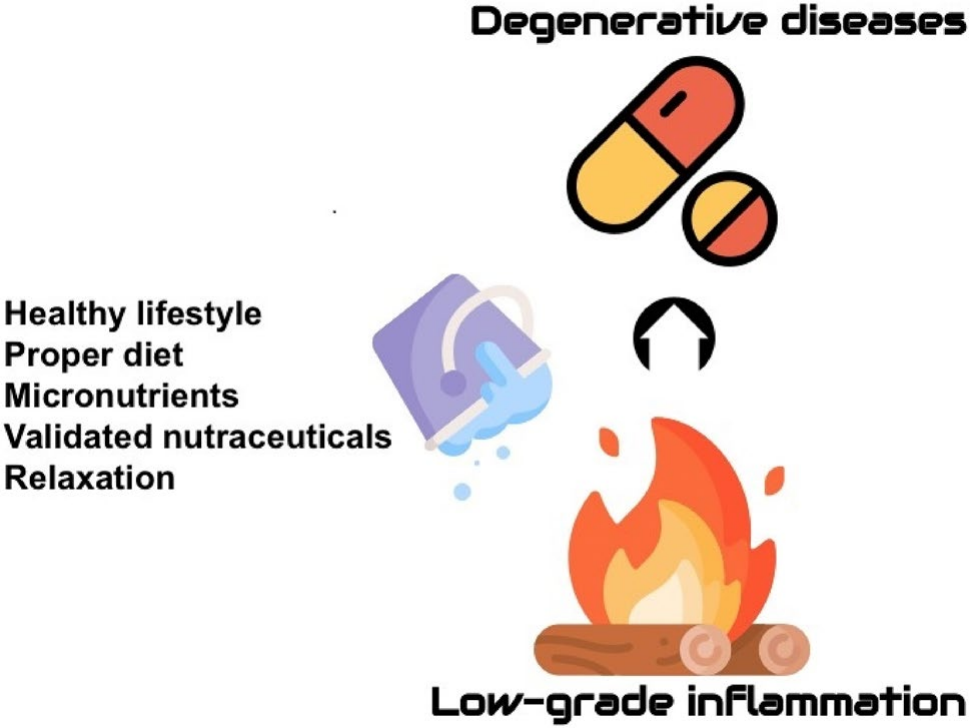
Examples of non-pharmacological interventions to lessen low-grade, chronic inflammation

Given the aforementioned evidence of the noxious role of low-grade inflammation in the etiology of several pathologies (many of which are associated with and exacerbated by aging [10]), the issue arises of how can we defuse it without always resorting to pharmacological treatment. Many lifestyle interventions are being investigated and are worth discussing (Fig. 2).

Even though it is at present unclear what the main triggers of low-grade inflammation are, the first line of defense against its deleterious effects is the adoption of a healthy lifestyle. This includes proper diet/nutrition, physical exercise, mediation, and reducing exposure to stressors [5, 10, 119]. Because one of the features of low-grade inflammation-associated disorders is an altered gut microbiota (often called dysbiosis) characterized by a reduced diversity with fewer butyrate-producing *Firmicutes* and more gram negative pathobionts [10, 98], attention should be paid to fibers and prebiotics, as well as other dietary components briefly outlined below.

Diets rich in plant foods have long being associated with better cardiovascular health, including lower incidence of cardiovascular disease and cancer. One notable example is the Mediterranean diet, whose healthful properties are now irrefutable. In particular, adherence to a Mediterranean-like diet with its high consumption of wild plants has been shown to decrease systemic markers of inflammation, namely C-reactive protein (CRP), interleukin (IL) 6, and homocysteine [18]. This reduced systemic inflammation translates, for instance, into augmented vascular compliance [37]. Consequently, basic researchers are concentrating their efforts on ascertaining which food items, e.g., cereals, fruits, vegetables, olive oil, and individual components, e.g., fibers, vitamins, and (poly)phenols are most responsible for the salubrious actions of plant-based diets. (Poly)phenols are secondary metabolites of plants, where the play important roles in resistance to microorganisms and insects, pigmentation, and organoleptic characteristics (odor and flavor) [120].

(Poly)phenols, namely ortho-diphenols, have been long mischaracterized as antioxidants. While this is true in a test tube, their very low bioavailability and reactivity impede direct free radical scavenging in vivo. Other mechanisms of action have been proposed to explain the healthful effects of (poly)phenol ingestion. The most investigated and agreed upon one is activation of stress-response pathways, namely as mediated by NRF2 (Nuclear factor erythroid 2-related factor 2). It must be underscored that, although there are plenty of in vitro studies strongly suggesting this hypothesis, in vivo evidence is lacking [25]. The biological activities of (poly)phenols, however, extend beyond their mere indirect antioxidant action and include modulation of inducible nitric oxide synthase and of cyclo- and lipooxygenases. Among the most studied ones, polyphenols from green tea, grapes, olives, and berries have been shown to decrease the production of inflammatory markers, e.g., leukotriene B_4_, in several systems [7, 26]. Mechanistically, modulation of the expression of inflammatory mediators includes inhibition of transcription factors such as NF-κB (nuclear factor kappa-light-chain-enhancer of activated B cells) and AP-1 (Activator protein 1), via the inhibition of protein kinases involved in signal transduction. In summary, the healthful effects of (poly)phenols observed in epidemiological and intervention studies are due to an array of activities, among which inhibition of inflammation likely plays a major role [120].

Increased intake of long-chain omega-3 polyunsaturated fatty acids (PUFAs) results in increased proportions of those fatty acids in cell membranes, including those of inflammatory cells [82]. The incorporation of EPA (Eicosapentaenoic acid) and DHA (Docosahexaenoic acid) into human inflammatory cells displaces arachidonic acid, leading to less substrate available for synthesis of the classic inflammatory eicosanoids, e.g., prostaglandin E_2_. In addition to altered eicosanoid production, omega-3 PUFAs affect inflammation and inflammatory processes via non-eicosanoid-mediated actions on cell signaling and gene expression [82]. Thus, EPA and DHA are considered to have anti-inflammatory effects. It should be noted, however, that not all trials confirmed this hypothesis. The lack of consistency may be related to differences in: duration of treatment; sample size; characteristics of the populations studied; background diet; dose of EPA + DHA used; chemical formulation (e.g., triglycerides vs. ethyl esters); and genetic differences among individuals studied [122].

Theoretically, omega-6 fatty acids, namely arachidonic acid, could be pro-inflammatory, being the precursors of eicosanoids. Indeed, biochemical theories posit the competitive relationship with omega-3 fatty acids, with which omega-6 should compete for some enzymatic activities, i.e., elongation and desaturation [121]. However, the majority of human studies do not confirm this biochemical hypothesis [20] and a systematic review of the literature did not actually conclude on any increase in inflammatory markers associated with the consumption of omega-6 in humans [36]. In summary, an increase in polyunsaturated (be them omega-3 or omega-6) fatty acids dietary intake, from fish, seed oils, vegetables, and nuts is associated with lower systemic inflammation and likely reduces the risk of degenerative diseases [121].

The interplay between inflammation and oxidative stress has not been clearly elucidated, although it appears that there is a bidirectional interaction between the two [10]. Hence, the anti-inflammatory effects of antioxidants such as the antioxidant vitamins and (poly)phenols are frequently tested together. Overall, data are not conclusive, but there is evidence of anti-inflammatory effects of vitamin E, lycopene, and astaxanthin, whereas, the activities of vitamin D as an anti-inflammatory agent are still equivocal [10].

Whereas acute inflammation is, to some degree, well-characterized and appropriately treatable with drugs, the many bidirectional interactions make low-grade inflammation difficult to disentangle and point to the highly complex biological processes involved with single-target interventions aimed at combating inflammation and its related morbidities, which may fail if they do not consider the underlying complex network of interactions. We also need validated and easy-to-measure biomarkers, e.g., microRNAs, galactosylated immunoglobulins G, circulating mitochondrial DNA, cytokines, etc. While research progresses, it is important to reiterate the message that adoption of a healthy lifestyle and diet greatly contributes to the lessening of chronic inflammation and its noxious corollaries.

## A nutrient reference value (NRV) process for bioactives: public health and healthy ageing

This section summarizes the findings of a December 4th, 2019 workshop examining a path for the establishment of a nutrient reference value (NRV), termed in the U.S. and Canada as a dietary reference intake (DRI), via the process adopted by the U.S. National Academies of Sciences, Engineering and Medicine (NASEM) [NOTE: formerly the U.S. Institute of Medicine with the acronym ‘IOM’] for eicosapentaenoic acid (20:5n-3; EPA) and docosahexaenoic acid (22:6n-3; DHA) that was recently published in detail elsewhere [91]. Herein, is the context and biochemical background as to how EPA and DHA differ from other nutrients for which there is already an accepted DRI and a proposed path forward by which one could consider EPA and DHA under a modified framework.

EPA and DHA can be synthesized from a variety of dietary precursors with alpha-linolenic acid (18:3n-3; ALA) being the most important from a nutritional perspective [79]. While there is a wide range of intakes for n-3 polyunsaturated fatty acids (PUFA), typical median North American intakes of EPA and DHA are about 100 mg per day while ALA intakes are many times higher at approximately 1600 mg per day [109]. Although there is some debate in the literature around the efficacy of EPA and DHA synthesis from ALA, clearly ALA can be converted to EPA and DHA [69] and maintain their blood levels at least to an extent [17, 123]. Unlike ALA, the absence of EPA and DHA from the diet does not produce classical signs of deficiency. Thus, the Institute of Medicine (IOM) has deemed that only ALA is essential in the diet and has established and an Adequate Intake (AI) level for ALA, adding that up to 10% of the AI for ALA can be consumed as EPA and/or DHA [57]. Importantly, because the AI for ALA (as well as the potential 10% contribution from EPA and DHA) was set at the median intake of an U.S. reference population where n-3 PUFA deficiency was non-existant, individual intakes below the AI do not relate to a prevalence of inadequacy. Furthermore, almost by definition, approximately half of the population does not achieve intakes at the level of the AI. Clearly, there are major challenges to establishing the essentiality of EPA and DHA in the context of classical nutrition deficiency, and this is an apparent source of frustration to those who would advocate for the beneficial effects of EPA and DHA in the diet beyond the prevention of deficiency-induced scaly dermatitis. Thus, a workshop was held to discuss updates to the DRI process and the science on EPA and DHA. Hereafter is a summary of the key concepts and we refer the reader to a recent publication for more details [91].

An approach for bioactives with potential health benefits when consumed in the diet, but that are not nutritionally essential, has been proposed [38, 70, 92]. Furthermore, the Joint Canada-US Dietary Reference Intakes Working Group recently reported that they will “… broaden the scope of the DRIs to better incorporate the concept of chronic disease risk reduction …” [72], an approach that was recently used by the NASEM for sodium and potassium [84].

To establish a DRI for EPA and DHA, it will be critical to establish their causality in terms of disease reduction. While a valid surrogate could be considered as a biomarker of disease risk, given the potential pluripotent effects of EPA and DHA it is unclear what this surrogate could be. While the roles of EPA and DHA in neurodevelopment, inflammation and cognitive decline were considered, the effects on cardiovascular disease and premature births are of note. From the perspective of nutrition, there exists a relatively large literature examining randomized controlled trials RCTs) of EPA and DHA supplementation on cardiovascular disease endpoints. Several recent meta-analyses of RCTs have suggested there is a reduction in CVD risk upon EPA and DHA supplementation and importantly one recent meta-analysis demonstrated a dose response [6, 54]. Nevertheless, most RCTs were conducted in subjects with established heart disease and extrapolation to healthy adults, as well as the quality of the evidence will need to be further considered. Another candidate area for establishing a DRI for EPA and DHA is the prevention of premature births. A recent meta-analysis reported robust decreases in the risk of premature and early premature births upon supplementation with EPA and DHA, by 11% and 42%, respectively. Importantly, the evidence was also deemed to be high quality [81]. Based on this evidence, the Australian Government Pregnancy Care Guidelines states “Advise pregnant women that supplementation with omega-3 long-chain polyunsaturated fatty acids (800 mg DHA and 100 mg EPA per day) may reduce their risk of preterm birth, if they are low in omega-3”. Importantly, future work establishing the dose response of plasma biomarkers of EPA and DHA intake to the risk of both CVD and premature birth will be important.

Since, the last IOM report setting an AI for ALA, knowledge about the relationship between EPA and DHA intake and disease risk has advanced along with improvements in biomarkers of intake [80, 89] and a general understanding of their metabolism [79]. While the new guiding principles of chronic disease reduction might be a viable path forward for setting a future DRI for EPA and DHA, it does not negate other approaches that may be biochemically based as is often used for amino acids, and further research is warranted.

## Conclusion

It is no surprise that the global population has and is increasing, now nearing 8 billion humans. Though the curve continues its upward path, there are projections that show by mid 21st Century, the global population will reach a peak and the transition will be to an older world, with the substantive increased proportion of older people, especially the 80 + subgroup, which is the fastest growing from 14 million in 1950 to 384 million now, and counting. For the most part, we are all living longer than our forebears…with many population subsets living 30–35 years longer than at the turn of the 20th Century. Unfortunately, as life expectancies have increased, a term referred to as the ‘Longevity Revolution’, there has not been a parallel increase in the ‘healthy life expectancy’ (defined as the period of life spent in good health, free from the chronic diseases and disabilities of ageing) [76, 132]. As discussed in our opening section, ‘population ageing’, i.e., the phenomenon that many are living into their 9th and 10th decade of life, is now being observed in the developing world with serious and often heart-breaking consequences as personal and national economics, social services, public health measures, and familial and community support, are all deteriorating or non-existent. These inequalities are a global concern, and the World Health Organization is capitalizing on the sustainable development goals to address these issues, including a shout to end hunger, and provide healthier nutritional options and opportunities.

One significant macronutrient category that is critical, though often overlooked as the person or population ages, is stressing the need for adequate protein and amino acid intake. Without sufficient protein intake, lean body mass decreases, as evidenced by decrements in muscle and bone mass. The term, ‘sarcopenia’, refers to this age-related phenomenon, and often is accompanied by a parallel increase in body fat mass, such that to the casual observer, weight and body morphology appear to be in stasis. Frailty is the logical consequence of the loss in musculature and the increase in bone fracture. Data demonstrate that there is an annual 3.6% reduction in muscle mass accompanied by a consequential lowing of habitual gait speed and decreased grip strength in men aged 83.4 ± 3.9 years. Addressing this issue, with appropriate nutritional strategies is important to allow a healthy life expectancy that can be a source of *joie* de *vivre* during whatever life expectancy we are able to achieve.

The bioactive constituents, omega-3 fatty acids can be obtained from the diet, but only if the consumer understands the sources and concentrations needed to achieve as robust a benefit as possible. Omega-3 fatty acids, primarily docosahexaenoic acid (DHA) are added to fortified foods, especially infant formulas, as studies demonstrated that without maternal milk DHA, the non-breast-fed infants had very little DHA in their frontal cortex, with plausible decrements to visual acuity and cognitive function. Subsequent randomized control trials have demonstrated benefits to DHA fortification and supplementation on not only cognitive function, but also term birth weights, body lean and fat mass ratios, blood pressure and body weight.

The pro-hormone, vitamin D has a plethora of physiological activities, however, its role in immunity is of utmost interest, not only every day, but maybe more so during a global pandemic. WHO notes that there is a high prevalence of low vitamin D status globally, and in the US, the Dietary Guidelines for Americans (DGA) for 2015–2020 indicates that people in the U.S. do not consume enough dietary fiber, vitamin D, calcium, and potassium. These under consumed nutrients, are considered nutrients of public health concern because low intakes are associated with poor health outcome(s) [116]. One of the U.S. Government’s most important responsibilities is to protect the health of the American public; and today, about half of all American adults—117 million people—have one or more preventable, chronic diseases, many of which are related to poor quality eating patterns and physical inactivity. Rates of these chronic, diet-related diseases continue to rise, and they come not only with increased health risks, but also at high cost. Vitamin D supplementation has been shown to decrease upper and acute respiratory tract infections, as well support immune function as suggested by incipient data within the context of the current SARS-CoV-2 virus infection.

As presented in this paper, inflammation and its sequelae are thought to play a decisive role in the onset and development of many degenerative diseases, such as atherosclerosis, cardiovascular disease, cancer, and neurodegeneration. Chronic low-grade inflammation, also termed ‘inflammaging’ is insidious and leads to cellular changes that become manifest in many of these diseases, often in old age, hence their effect on the quality and quantity of one’s healthy life expectancy. There is possibly a bidirectional interchange between oxidative stress and inflammation, such that proven antioxidant and (poly)phenolic nutrients may play a role in lessening inflammation and inflammaging.

The end goal for this paper and the examples presented, is to establish or at least propose for regulatory consideration, public health goals to alleviate or at least ameliorate the scourges of debilitating health as one approaches the limit of our specie’s lifespan. The recommended beneficial regimens, including increased protein and amino acids, omega-3 fatty acids, Vitamin D at doses above the RDA, and (poly)phenols/antioxidants, needs more research. Astute consumers could evaluate the personal advantages to adopting one or more of these academic recommendations, and the data would suggest a longer life in good health for that individual, though one would never know how many years of benefit were objectively added. On a population basis, true improvement could be quantitated if sufficient individuals were to truly adopt one or more of these proposals, but without governmental public health recommendations or ingraining these counsels into dietary guidelines, school lunch programs, social service feeding plans, etc., that population ‘clinical trial’ will not commence.

Society’s response to population aging will require a vision to harness the years spent in ‘good health’ (e.g., the healthy life expectancy) to those hoped for extra years of life. In essence not only more years to life but also more life to years. A fundamental transformation in public policies and institutions is required to ensure a future that celebrates diversity yet narrows health inequities, within and across countries. “*Life should not be a journey to the grave with the intention of arriving safely in a pretty and well preserved body, but rather to skid in broadside in a cloud of smoke, thoroughly used up, totally worn out, and loudly proclaiming ‘Wow! What a Ride*!’” [42].

## Footnotes

1 The five cardinal signs of inflammation: rubor (flusing), tumor (swelling), calor (fever), dolor (pain), and function lesa (loss of function) from Galen and Celsus [133].
